# Characterization of Novel *PHEX* Variants in X-linked Hypophosphatemic Rickets and Genotype-PHEX Activity Correlation

**DOI:** 10.1210/clinem/dgae120

**Published:** 2024-03-05

**Authors:** Huixiao Wu, Hui Ying, Wanyi Zhao, Yan Sun, Yanzhou Wang, Xinyu Chen, Guimei Li, Yangyang Yao, Shuo Xu, Tianyou Li, Li Fang, Xiaoqing Sun, Ning Wang, Jin Xu, Qingbo Guan, Weibo Xia, Li Wang, Ling Gao, Jiajun Zhao, Chao Xu

**Affiliations:** Department of Endocrinology and Metabolism, Shandong Provincial Hospital Affiliated to Shandong First Medical University, Jinan 250021, Shandong, China; Department of Endocrinology and Metabolism, Shandong Provincial Hospital, Cheeloo College of Medicine, Shandong University, Jinan 250021, Shandong, China; Institute of Endocrinology, Shandong Academy of Clinical Medicine, Jinan 250021, Shandong, China; Shandong Clinical Medical Center of Endocrinology and Metabolism, Jinan 250021, Shandong, China; Department of Endocrinology and Metabolism, Shandong Provincial Hospital Affiliated to Shandong First Medical University, Jinan 250021, Shandong, China; Department of Endocrinology and Metabolism, Shandong Provincial Hospital, Cheeloo College of Medicine, Shandong University, Jinan 250021, Shandong, China; Institute of Endocrinology, Shandong Academy of Clinical Medicine, Jinan 250021, Shandong, China; Shandong Clinical Medical Center of Endocrinology and Metabolism, Jinan 250021, Shandong, China; Department of Endocrinology and Metabolism, Shandong Provincial Hospital Affiliated to Shandong First Medical University, Jinan 250021, Shandong, China; Department of Endocrinology and Metabolism, Shandong Provincial Hospital, Cheeloo College of Medicine, Shandong University, Jinan 250021, Shandong, China; Institute of Endocrinology, Shandong Academy of Clinical Medicine, Jinan 250021, Shandong, China; Shandong Clinical Medical Center of Endocrinology and Metabolism, Jinan 250021, Shandong, China; Department of Pediatric, Shandong Provincial Hospital Affiliated to Shandong First Medical University, Jinan 250021, Shandong, China; Department of Pediatric Orthopedics, Shandong Provincial Hospital Affiliated to Shandong First Medical University, Jinan 250021, Shandong, China; Department of Endocrinology and Metabolism, Shandong Provincial Hospital Affiliated to Shandong First Medical University, Jinan 250021, Shandong, China; Department of Endocrinology and Metabolism, Shandong Provincial Hospital, Cheeloo College of Medicine, Shandong University, Jinan 250021, Shandong, China; Institute of Endocrinology, Shandong Academy of Clinical Medicine, Jinan 250021, Shandong, China; Shandong Clinical Medical Center of Endocrinology and Metabolism, Jinan 250021, Shandong, China; Department of Pediatric, Shandong Provincial Hospital Affiliated to Shandong First Medical University, Jinan 250021, Shandong, China; Department of Pediatric Orthopedics, Shandong Provincial Hospital Affiliated to Shandong First Medical University, Jinan 250021, Shandong, China; Department of Medical Imaging, Shandong Provincial Hospital Affiliated to Shandong First Medical University, Jinan 250021, Shandong, China; Department of Pediatric Orthopedics, Shandong Provincial Hospital Affiliated to Shandong First Medical University, Jinan 250021, Shandong, China; Department of Endocrinology and Metabolism, Shandong Provincial Hospital Affiliated to Shandong First Medical University, Jinan 250021, Shandong, China; Department of Endocrinology and Metabolism, Shandong Provincial Hospital, Cheeloo College of Medicine, Shandong University, Jinan 250021, Shandong, China; Institute of Endocrinology, Shandong Academy of Clinical Medicine, Jinan 250021, Shandong, China; Shandong Clinical Medical Center of Endocrinology and Metabolism, Jinan 250021, Shandong, China; Institute of Endocrinology, Shandong Academy of Clinical Medicine, Jinan 250021, Shandong, China; Shandong Clinical Medical Center of Endocrinology and Metabolism, Jinan 250021, Shandong, China; Institute of Endocrinology, Shandong Academy of Clinical Medicine, Jinan 250021, Shandong, China; Shandong Clinical Medical Center of Endocrinology and Metabolism, Jinan 250021, Shandong, China; Department of Endocrinology and Metabolism, Shandong Provincial Hospital Affiliated to Shandong First Medical University, Jinan 250021, Shandong, China; Department of Endocrinology and Metabolism, Shandong Provincial Hospital, Cheeloo College of Medicine, Shandong University, Jinan 250021, Shandong, China; Institute of Endocrinology, Shandong Academy of Clinical Medicine, Jinan 250021, Shandong, China; Shandong Clinical Medical Center of Endocrinology and Metabolism, Jinan 250021, Shandong, China; Department of Endocrinology and Metabolism, Shandong Provincial Hospital Affiliated to Shandong First Medical University, Jinan 250021, Shandong, China; Department of Endocrinology and Metabolism, Shandong Provincial Hospital, Cheeloo College of Medicine, Shandong University, Jinan 250021, Shandong, China; Institute of Endocrinology, Shandong Academy of Clinical Medicine, Jinan 250021, Shandong, China; Shandong Clinical Medical Center of Endocrinology and Metabolism, Jinan 250021, Shandong, China; Department of Endocrinology, Key Laboratory of Endocrinology, Ministry of Health, Peking Union Medical College Hospital, Chinese Academy of Medical Sciences & Peking Union Medical College, Beijing 100730, China; Independent Researcher, Tucson, AZ 85705, USA; Department of Endocrinology and Metabolism, Shandong Provincial Hospital Affiliated to Shandong First Medical University, Jinan 250021, Shandong, China; Department of Endocrinology and Metabolism, Shandong Provincial Hospital, Cheeloo College of Medicine, Shandong University, Jinan 250021, Shandong, China; Institute of Endocrinology, Shandong Academy of Clinical Medicine, Jinan 250021, Shandong, China; Shandong Clinical Medical Center of Endocrinology and Metabolism, Jinan 250021, Shandong, China; Department of Endocrinology and Metabolism, Shandong Provincial Hospital Affiliated to Shandong First Medical University, Jinan 250021, Shandong, China; Department of Endocrinology and Metabolism, Shandong Provincial Hospital, Cheeloo College of Medicine, Shandong University, Jinan 250021, Shandong, China; Institute of Endocrinology, Shandong Academy of Clinical Medicine, Jinan 250021, Shandong, China; Shandong Clinical Medical Center of Endocrinology and Metabolism, Jinan 250021, Shandong, China; Department of Endocrinology and Metabolism, Shandong Provincial Hospital Affiliated to Shandong First Medical University, Jinan 250021, Shandong, China; Department of Endocrinology and Metabolism, Shandong Provincial Hospital, Cheeloo College of Medicine, Shandong University, Jinan 250021, Shandong, China; Institute of Endocrinology, Shandong Academy of Clinical Medicine, Jinan 250021, Shandong, China; Shandong Clinical Medical Center of Endocrinology and Metabolism, Jinan 250021, Shandong, China

**Keywords:** genotype–PHEX activity correlation, mouse model, PHEX, XLHR, variant

## Abstract

**Background:**

X-linked hypophosphatemia (XLHR) is the most common genetic form of hypophosphatemic rickets (HR), which is caused by phosphate regulating endopeptidase homolog X-linked (PHEX) gene mutation. At present, the genotype–phenotype relationship of XLHR and the pathogenic role of PHEX are not fully understood.

**Methods:**

In this study, we summarized clinical features in a new cohort of 49 HR patients and detected 16 novel PHEX and 5 novel non-PHEX variants. Subsequently, we studied the pathogenesis of new variants by protein expression, glycosylation analysis, subcellular localization, and endopeptidase activity.

**Results:**

The results showed that missense variants (Q189H and X750R) slightly reduced protein expression without obviously altering protein length and localization, whereas truncating variants significantly impaired the synthesis of PHEX and produced a shorter immature protein in cells. Interestingly, no evident correlation was observed between mutation types and clinical phenotypes. However, when we analyzed the relationship between PHEX activity and serum phosphorus level, we found that patients with low PHEX activity tended to have severe hypophosphatemia and high rickets severity score. Following this observation, we established 2 new knock-in XLHR mouse models with 2 novel Phex variants (c.T1349C and c.C426G, respectively) using CRISPR/Cas9 technology. Both mouse models demonstrated clinical manifestations of XLHR seen in patients, and PhexC426G mice showed more severe phenotype than PhexT1349C mice, which further confirmed the rationality of genotype–PHEX enzymatic activity correlation analysis.

**Conclusion:**

Therefore, our findings demonstrated that novel PHEX variants could disrupt protein function via affecting protein synthesis, post-translational modification, cellular trafficking, and catalytic activity. Our study facilitates a better understanding of XLHR pathogenic mechanism and PHEX activity-phenotype correlation, which is of crucial importance for future diagnosis and treatment of XLHR.

Hypophosphatemic rickets (HR), characterized by hypophosphatemia and skeletal dysplasia, is a rare metabolic bone disease in children mainly due to genetic mutations. HR is often accompanied by early onset, obvious clinical symptoms, and a high incidence of disability (1). HR includes several subtypes such as X-linked hypophosphatemic rickets [XLHR; Online Mendelian Inheritance in Man (OMIM) #307800], which accounts for more than 80% of HR; autosomal dominant hereditary hypophosphatemic rickets [OMIM #193100: fibroblast growth factor 23 (FGF23)], autosomal recessive hereditary hypophosphatemic rickets (ARHR) [OMIM #241520: ARHR1 (DMP1); OMIM #613312: ARHR2 (ENPP1); OMIM #611061: ARHR3 (FMA20C)], hereditary hypophosphatemic rickets with hypercalciuria (OMIM #241530), and Dent disease (2). XLHR is caused by mutations in the phosphate-regulating gene with homologies to endopeptidases on the X chromosome (*PHEX*; OMIM #300550) (3), which is characterized by hypophosphatemia, aberrant vitamin D metabolism, growth retardation, and bone mineralization defects and is associated with a pathological elevation of FGF23 (4). The phosphate-regulating gene with homologies to endopeptidases on the X chromosome (PHEX) gene comprises 22 exons and encodes a transmembrane endopeptidase belonging to the type II integral membrane zinc-dependent endopeptidase family. PHEX is composed of 749 amino acids with 3 domains: a short cytoplasmic N-terminal region (amino acids 1-20), a single transmembrane domain (amino acids 21-37), and a long extra-cytoplasmic domain (amino acids 38-749), which contains zinc-binding motifs as the active site of the enzyme. Up to now, there have been more than 640 mutations in the *PHEX* gene reported in Human Gene Mutation Database (HGMD) resulting in XLHR, and only a limited number of mutations have been evaluated for their pathogenic effects. Moreover, PHEX acts as an endopeptidase, and there has been no study investigating the correlation between the catalytic activity of PHEX and patients’ clinical manifestations.

Previous studies have explored the genotype–phenotype correlation of XLHR. The majority of these studies demonstrated that there was no obvious correlation between disease severity and the type or location of *PHEX* mutations from a clinical perspective without functional studies (5-9). In a phenotype analysis of 31 XLHR patients, a trend toward more severe skeletal disease was found in patients with truncating mutations and a family history (9). Another genotype–phenotype correlation study of 36 patients revealed that patients with clearly deleterious *PHEX* mutations had lower tubular reabsorption of phosphate and 1,25(OH)_2_ D levels (10). Apparently, results from these genotype–phenotype analysis were inconsistent. One possible explanation would be that PHEX was a peptide enzyme and each *PHEX* variant might have a different effect on its activity. More recently, a Japanese study conducted a genotype–phenotype relationship analysis using 3D modeling and showed that the zinc-binding site and cavity in PHEX can play a critical role in its function. These findings provide new clues for investigating the function of PHEX and the pathogenesis of XLHR (11). Therefore, it is rational to perform a genotype–phenotype analysis from the perspective of enzyme activity of PHEX. Nevertheless, there are as yet no studies to explore the correlation between the PHEX activity and the phenotype of XLHR.

In the present study, we established a clinical HR cohort. We enrolled 49 clinically diagnosed HR patients in which 44 patients had XLHR and 5 patients had other types of HR. From this cohort, we identified 16 *PHEX* variants and 5 non-*PHEX* variants that have not been reported before. We performed genotype–phenotype and enzyme activity–phenotype correlation analyses and evaluated the pathogenic role of novel *PHEX* variants through in vitro experiments. We also selected 2 missense variants causing pronounced phenotypes (c.T1349C and c.C426G) to establish 2 new knock-in mouse lines (*Phex*^T^1349^C^ and *Phex*^C426G^). Both hemizygous male and heterozygous female mice exhibited typical clinical manifestations of XLHR, including hypophosphatemia and skeletal dysplasia (rickets/osteomalacia). These findings highlight the valuable addition of novel *PHEX* variants and 2 novel mouse models to the existing mouse models of XLHR, which provided new pathogenic insight into the role of PHEX in bone mineralization. Our study also suggests that PHEX enzymatic activity may serve as a potential new efficacious indicator to predict disease severity and prognosis of XLHR.

## Materials and Methods

### Study Subjects

The human research was conducted according to the guidelines stated in the Declaration of Helsinki. The Institutional Review Board approved the research protocol, and written informed consent was obtained from the parents of the participants. We retrospectively analyzed the clinical manifestations of 49 unrelated patients with HR whose diagnosis was verified by genetic analysis in Shandong Provincial Hospital from 2016 to 2020. All immediate family members received specific physical and laboratory examinations, and the peripheral blood specimens were collected from each member for genetic analysis.

### Clinical and Biochemical Data Collection

All data were collected from patients at the first visit without any treatment. The clinical data included age of disease onset, age at diagnosis, height, family history, clinical symptoms, and rachitic signs. The biochemical data included fasting serum phosphorus (Pi), calcium (Ca), alkaline phosphatase (ALP), PTH, and 25-hydroxyvitamin D [25-(OH)D], which were examined in clinical laboratory in our hospital. FGF23 and α-Klotho levels were measured using human FGF23 and soluble α-Klotho Elisa kits (SenBeiJia Biological Technology Co., Ltd., RRID: AB_3076183 and RRID: AB_3076184). Height was presented as SD score (SDS) using standardized growth charts for Chinese children and adolescents aged 0 to 18 years (2). The Rickets Severity Score (RSS) was evaluated for some XLHR patients whose X-ray results were available using the method of Thacher (3). It is a 10-point scale, where 10 represents the most severe category and 0 represents the normal condition. Radiographs were read and scored by 2 independent physicians, and the mean score was used.

### Mutation Analysis

Genomic DNA was isolated from peripheral blood leukocytes using the QIAamp DNA Mini Kit (Qiagen, Germany) following the manufacturer's instructions. Whole-exome sequencing was performed on DNA from peripheral blood. After genomic DNA fragmentation, paired-end adaptor ligation, amplification, and purification, the human exons were captured by using the SeqCap EZ Med Exome Enrichment Kit (Roche NimbleGen, USA). The DNA library was generated by postcapture amplification and purification and then sequenced on the Illumina HiSeq sequencing platform. Sequence data alignment to the human genome reference (hg19) and variant-calling were performed with NextGene V2.3.4 software to obtain the coverage and mean read depth of the target regions. The average coverage of the exome was >100×, which permitted the examination of the target region with enough depth to exactly match >99% of the target exome. To ensure the accuracy of data analysis, mutations with low coverage in the target area were filtered out.

Additionally, annotation information, including the conservation of nucleotide bases and amino acids; predictions of biological functions; the frequency in normal populations (Genome Aggregation Database, Trans-Omics for Precision Medicine, the Exome Aggregation Consortium); and data from the HGMD, Clinvar, and OMIM databases was evaluated by using NextGene V2.3.4 and our in-house scripts. A variant was recognized as a mutant when it was not found in dbSNP (http://www.ncbi.nlm.nih.gov/snp/), in the exome variant server (http://evs.gs.washington.edu/EVS/), in the ensemble database, and in 500 Chinese controls, or, alternatively, the allele frequency was found to be less than 0.001 in the database. Pathogenic variants were determined according to the Standards and Guidelines for the Interpretation of Sequence Variants published by the American College of Medical Genetics and Genomics in 2015 with the Human Genome Variation Society nomenclature.

We use whole-exome sequencing to detect candidate variants. We then verified the detected pathogenic or suspected pathogenic variants by Sanger sequencing and ensured that the coverage of the gene coding sequence reached 100%. Using Primer3 version 1.1.4 (http://www.sourceforge.net) and GeneDistiller 2014 (http://www.genedistiller.org/), tagged sequencing primers of *PHEX* were designed. Polymerase chain reaction (PCR) was performed in a 50 μL system including 4 μL genomic DNA, 1 μL forward and reverse primers, 5 μL 10 × PCR buffer, 4 μL dNTPs, and 0.3 μL Taq Hot Start (Takara Bio, Ohtsu, Japan). The PCR conditions were an initial denaturation step (95 °C for 5 minutes), followed by 40 cycles of denaturation (95 °C for 30 seconds), annealing (65 °C for 30 seconds), and elongation (72 °C for 30 seconds). Amplicons were sequenced using an ABI 3730 system (Applied Biosystems, Foster City, CA, USA), and sequence analysis was performed using the autoassembler software Chromas 2.6.6 (Technelysium Pty Ltd., www.technelysium.com.au/chromas.html) and visual inspection.

### Bioinformatic Assays

To test whether the mutation was benign or malignant, software PolyPhen-2 (http://genetics.bwh.harvard.edu/pph2/) and Mutation Taster (http://www.mutationtaster.org/) were performed to predict potential effect. Multiple sequence alignment was measured by using Clustal Omega (https://www.ebi.ac.uk/Tools/msa/clustalo/). Tertiary structure model of the wild-type (WT) and mutant PHEX was predicted from ITASSER (https://zhanglab.ccmb.med.umich.edu/I-TASSER/). All the models were presented on the software of PyMOL (version 1.3).

### Functional Analysis

#### Immunoblot analysis

Bone tissues from normal control and XLHR patients were lysed in radioimmunoprecipitation assay buffer supplemented with protease and phosphatase inhibitors. Protein lysates (60 μg) were separated using 10% SDS-PAGE and transferred onto polyvinylidene fluoride membranes (Millipore). The membranes were blocked with TBST containing 5% slim milk for 1 hour at room temperature and then incubated with primary antibodies against PHEX (1:1000, Proteintech, RRID:AB_2882259), heat shock protein 90 (1:1000, Proteintech, RRID:AB_2120924), Tubulin (1:7500, Proteintech, RRID:AB_2210206), and GAPDH (1:7500, Proteintech, RRID:AB_2263076) overnight at 4 °C, after which they were incubated with secondary antibodies (Proteintech, RRID: AB_2722564&AB_2890995) for 1 hour at room temperature. Immobilon Western HRP Substrate Peroxide Solution (Millipore, USA) was used for membrane development.

### Plasmid Construction of PHEX and Cell Transfection

The full length of the major transcript of human PHEX (transcript ID: NM_000493.4) was synthesized and cloned into the transient overexpression vector GV141 (GeneChem, China), using the restriction enzymes XhoI and BamHI (NEB, USA). Each mutant *PHEX* was created by the Quickchange mutagenesis kit (Stratagene, La Jolla, CA, USA) according to the manufacturer's instructions. The sequence of each variant plasmid was confirmed by Sanger sequencing. Each plasmid was transiently transfected into HEK293 cells using Lipofectamine3000 (L3000-015, Invitrogen, USA). The whole cell lysates were collected for protein extraction 48 hours after transfection. After lysing the cells using radioimmunoprecipitation assay buffer with proteinase inhibitors (Invitrogen), the proteins were separated with 10% SDS-PAGE after denaturation and then analyzed by Western blotting.

### Immunofluorescence Assay

Forty-eight hours after transfection, cells were grown on glass coverslips and cell culture dishes were fixed with 4% paraformaldehyde, permeabilized with 0.5% Triton X-100, and blocked for 1 hour in 5% fetal bovine serum. Immunostaining was accomplished with anti-PHEX (1:200; Biorbyt, RRID: AB_3076182) overnight at 4 °C. Species-specific Alexa Fluor 488 secondary antibodies (Thermo Fisher Scientific, RRID:AB_2633275 and RRID:AB_2633280) were used at 1:1000 at room temperature for 1 hour. Anti-Na+/K+-ATPase (1:200, Abcam, RRID:AB_991679), a plasma membrane marker, was used to observe cellular localization of WT and mutant PHEX proteins. Nuclei were visualized by 4′6-diamidino-2-phenylindole (blue). Protein localization was observed by fluorescence microscopy (Carl Zeiss, Germany).

### Deglycosylation Analysis

For endo H digestion, WT and the variant PHEXs in the whole cell extracts were boiled for 10 minutes in 1× Glycoprotein Denaturing Buffer and incubated with endo H for 1 hour at 37 °C according to the manufacturer's recommendations (New England Biolabs). For the PNGase F digestion, the cell extracts were first incubated with 1×GlycoBuffer and mixed with PNGase F gently, then reacted with PNGase F at 37 °C for 5 hours according to the manufacturer's recommendations (New England Biolabs). Digestion products were fractionated on SDS-PAGE and subjected to immunoblot analysis as described earlier.

### Construction and Expression of a Soluble, Secreted Form of PHEX

To test the activity of PHEX enzyme both in WT and *PHEX* substitutions groups, a plasmid with a soluble and secreted form of PHEX, also known as secPHEX, was constructed as previously described (12). After the same method of transfection in HEK293 cells described earlier, whole cell lysates and culture medium were collected for cell PHEX and secPHEX extraction. Cell PHEX extraction was the same as previously mentioned, and the method of secPHEX extraction was performed according to the previous study (13). Purified WT and mutant secPHEX were used for activity analysis.

### Enzymic Activity of WT and Mutant secPHEX

SecPHEX activity in different groups was assayed in 0.01 M Bis/Tris buffer (pH 5.5) with 150 mM NaCl routinely used to test enzymic activity. Purified secPHEX both in WT and mutant groups were quantified by Western blotting. Abz-GFSDYK(Dnp)-OH (30 µM), a peptide containing a putative secPHEX cleavage site, was used as a substrate to interact with secPHEX in 0.01 M Bis/Tris buffer at 37 °C. After interacting for 30 minutes, the fluorescence was finally measured by fluorimeter (Cary Eclipse, USA) at λ (emission) = 420 nm and λ (excitation) = 320 nm. For heat stability studies, secPHEX proteins were incubated at the different temperatures (25, 40, and 55 °C) for 10 minutes, and the samples were placed on ice before assaying for activity as described previously.

### *Phex* Cas9-KI Mice Generation

All the animal experimental protocols were approved by the Animal Care Committee of School of Medicine, Shandong Provincial Hospital. Animals with novel missense variant in the *Phex* gene (hemizygous males: C426G/Y, T1349C/Y; heterozygous females: C426G/+, T1349C/+; homozygous females: C426G/C426G, T1349C/T1349C) were generated from Gempharmatech Corp. in China via CRISPR/Cas9 technology with a C57BL/6J background. Our breeding strategy generated heterozygous, homozygous, and WT females as well as hemizygous and WT males. Mice were caged in groups of 3 to 5, maintained on a 12-hour dark/light cycle, and provided standard Global 18% protein rodent chow diet (Beijing Keaoxieli Feed Co. Ltd; 1-1.8% Ca, 0.6-1.2% Pi) and water ad libitum. Mice at 12 weeks of age were used for phenotypic analysis.

Blood samples were collected by eyeball extirpation, then centrifuged to collect serum and stored at −80 °C for further biochemical analysis. Left femurs were collected, wrapped in saline-soaked gauze, and then frozen for a 3-point bending mechanical test. Right femurs were fixed in paraformaldehyde and used for micro-computed tomography (CT) analysis and bone histology.

### Genotyping of Mice

DNA was extracted from tail tissue at 3 weeks of age using a TIANamp Genomic DNA Kit following standard procedures (TIANGEN, China). Then PCR was performed as follows: 95 °C for 5 minutes, 20 cycles of 98 °C for 30 seconds, 65 °C (−0.5 °C/cycle) for 30 seconds, and 72 °C for 45 seconds, 20 cycles of 98 °C for 30 seconds, 55 °C for 30 seconds, and 72 °C for 45 seconds, and a final extension of 72 °C for 5 minutes. The products were then analyzed on a 3730xl DNA analyzer (ABI, USA) to determine whether the mutation site had been inherited. The specific mutation was identified by direct sequencing of *Phex* exons from amplified genomic DNA.

### Faxitron X-ray

X-ray pictures of whole mice were taken using a DR uDR588i from United Imaging Corp. in China. X-ray images were taken under constant conditions (50 kV, 40 ms). Analysis of body and femur lengths were determined from the DICOM files using the RadiAnt DICOM Viewer software (Poznan, Poland).

### Biochemistry Analysis

Pi, Ca, creatinine, blood urea nitrogen, AST, ALT, and ALP levels were measured using an automatic biochemical analyzer (BS-830, Mindray, China). FGF23 levels were measured using mouse FGF23 Elisa kits (SenBeiJia Biological Technology Co., Ltd., RRID: AB_3076185) following the manufacturer's instruction. Urine samples were collected at 24 hours, and renal phosphate excretion was determined. Renal phosphate excretion is expressed as fractional phosphate excretion (FPE_PO4_). FPE_PO4_ is calculated by urine phosphate concentration times serum creatinine concentration divided by serum phosphate concentration times urine creatinine concentration.

### Micro-CT (μCT) Analysis

Right femurs of the mice were stored in paraformaldehyde before being scanned. Scanning parameters were 70 kVp and 114 µA, with a 250 ms integration time and a 15.6 µm isotropic voxel size (vivaCT60, Scanco Medical). Cortical geometry was measured in the middle 100 slices of the femoral diaphysis. Primary cortical parameters included cortical bone area/cortical total area and cortical thickness. Trabecular bone architecture was analyzed from the distal 120 slices of the total femoral length to the distal growth plate. Primary trabecular parameters mainly included trabecular number and trabecular spacing. All parameters are reported using conventional nomenclature (14). Group sample sizes for all µCT analyses ranged from n = 3 to 7 mice.

### Mechanical Test

Left femurs of the mice were thawed in saline prior to 3-point bending. Femurs were loaded to fracture in the anterior-posterior direction. A lower support span length of 7 mm was used for mice. A load rate of 0.1 mm/s and a preload of ∼0.5 N were applied to each bone to prevent shifting during testing (3230 SERIES III, WATERS Corp.). Load-displacement curves were used to calculate the peak load and bending stiffness. Group sizes for mechanical testing ranged from n = 3 to 7 mice.

### Statistical Analysis

Statistical analysis was performed by SPSS 19.0 software package (SPSS Inc., Chicago, IL, USA). The Kolmogorov–Smirnov test was used to determine the distribution of continuous variables. Continuous variables with normal distribution were given as mean ± SD and compared by independent samples Student's *t*-test, whereas those with nonnormal distribution were given as median (25th, 75th percentiles) and compared by the Mann–Whitney U-test. The results were considered statistically significant when the *P*-value <0.05.

## Results

### Clinical Characteristics

We enrolled a total of 44 XLHR patients (22 males, 22 females) and 2 patients with ARHR2, 1 with autosomal dominant hereditary hypophosphatemic rickets, 1 with hereditary hypophosphatemic rickets with hypercalciuria, and 1 with type 2 Dent disease. There were 25 familial cases, which included 24 sporadic cases and only 1 patient inherited *PHEX* variant from her father. The clinical features of XLHR patients are summarized briefly in Table 1. For XLHR patients, the median age of disease onset was 15 months (12, 24; 25th, 75th percentile). The average height SDS of diagnosis was −2.37, indicating short stature of those patients. A majority of the patients (40/44) had genu varum while only 9% (4/44) had genu valgum. Serum phosphorus was below the lower limit of the normal range. Serum ALP, FGF23, and αKlotho were obviously higher than the upper limit of the normal range while serum PTH was slightly elevated and 25-(OH)D was in normal range.

**Table 1.** Characteristics of 44 patients with X-linked hypophosphatemic rickets

	Patients (n = 44)^*a*^	Reference range
Sex (male/female)	22/22	/
Familial/sporadic cases	21/23	/
Age of onset (months)	15 (12, 24)	/
Height at diagnosis (SDS)	−2.37 ± 2.11	/
Lower limb deformity		
Genu varum	40	/
Genu valgum	4	/
Serum Pi (mmol/L)	0.81 ± 0.15	1.55-2.65 (<1 yr)^*b*^ 1.25-2.1 (1-3 yr) 1.2-1.8 (4-11 yr) 0.95-1.75 (12-15 yr) 0.9-1.5 (>15 yr)
Serum ALP (U/L)	710.18 ± 262	98-532 (< 6 m)^*c*^ 106-420 (6m-1 yr) 128-432 (1-2 yr) 143-406 (2-9 yr) 146-500 (9-12 yr) M: 160-610, F: 81-454 (12-14 yr) M: 82-603, F: 63-327 (14-15 yr) M: 64-443, F: 52-215 (15-17 yr) M: 51-202, F: 43-130 (17-18 yr)
Serum PTH (pg/mL)	79.54 ± 37.1	15-65
Serum 25-(OH)D (ng/mL)	32.22 ± 19	47.7-144
iFGF23 (ng/L)	1168.84 (971.13, 1312.36)	235.3 ± 87.2^*d*^
αKlotho (ng/L)	15.45 ± 5.86	876.5 ± 312.4

Abbreviations: 25-(OH)D, serum 25-hydroxyvitamin D; ALP, serum alkaline phosphatase; Ca, serum calcium; F, female; iFGF23, intact fibroblast growth factor 23; M, male; m, month(s); Pi, serum phosphate; SDS, SD score; yr, year(s).

^*a*^The results were the baseline diagnostic values of the patients.

^*b*^Serum phosphate reference ranges according to age subgroups (15).

^*c*^Serum alkaline phosphatase reference ranges at the central laboratory of Shandong Provincial Hospital.

^*d*^Reference range for iFGF23 was determined in our laboratory from healthy controls (Mean ± 2SD).

### Genetic Analysis

**Figure 1.**
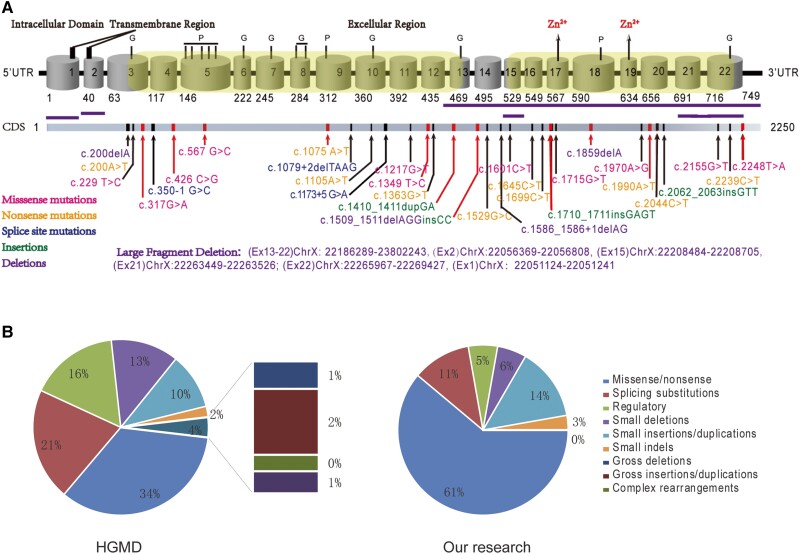
Schematic representation of PHEX and distribution of PHEX mutations. (A) Exon structure of the PHEX gene. The location of 36 PHEX mutations identified in this study were shown. Variants in red correspond to novel variants. The amino acid numbers defining each domain are shown below the exon structure. Major domains and modification sites of PHEX are labeled above the exons. Gross deletions are marked with horizonal lines of different colors. (B) Frequency distribution of PHEX mutations recorded in the HGMD (left) and in our XLHR cohort (right). Abbreviations: G, glycosylation site; HGMD, Human Gene Mutation Database; P, phosphorylation site; PHEX, phosphate regulating endopeptidase homolog X-linked; XLHR, X-linked hypophosphatemia.

To identify new genetic variants, whole-exome sequencing and multiplex ligation-dependent probe amplification were performed in all available immediate family members. As shown in Fig. 1A, 44 XLHR patients harbored 36 different *PHEX* variants, respectively, involving 20 missense/nonsense substitutions, 2 small deletions, 5 gross deletions, 4 splicing variants, 4 small insertions, and 1 complex arrangement. Among all *PHEX* variants, 16 variants had not been reported before, including 7 missense/nonsense variants (c.317G > A, c.426 C > G, c.567 G > C, c.1075A > T, c.1349 T > C, c.1990A > T, c.2248T > A), 5 gross deletions (EX1del, EX2del, EX15del, EX13-22del, EX21-22del), 2 small insertions (c.1410_1411dupGA, c.1711insGAGT), 1 small deletion (c.1859delA), and 1 complex arrangement (c.1509_1511delTGGinsCC). Mutation frequencies of different *PHEX* mutation types in HGMD and our cohort are, respectively, shown in Fig. 1B. Although the percentages of each mutation type varied in these 2 cohorts, it was evident that point mutation constituted the majority of mutations.

In order to analyze the potential effects of novel variants on the protein function, we summarized all novel *PHEX* and non-*PHEX* variants, the corresponding amino acid sequences as well as domains of mutant proteins altered by those variants (Supplementary Figs. S1-S3) (16). As for 5 gross deletions, all of them were involved in the highly conserved regions or in the critical domains like transmembrane domain and zinc-binding or catalytic sites that have an important impact on the integrity and function of the protein (Supplementary Fig. S4) (16). For other mutation sites, most of them were also located in several key regions of the structural domain, such as glycosylation sites, phosphorylation sites, and zinc-finger binding sites, which were crucial for post-translational modification of proteins and substrate binding. Of note, except for 2 large deletions situated in exon 1 and 2, all other mutations occurred in the extracellular segment, suggesting the importance of the extracellular domain for the function of PHEX.

### Bioinformatic Analysis

Using in silico analysis, we predicted the disease-causing effects of these novel variants and found that all missense variants affected highly conserved amino acids in diverse biological species by multiple sequence alignment. Concurrently, Mutation Taster and PolyPhen-2 (Supplementary Fig. S5) (16) also suggested that the missense sequence alterations in the conserved residues probably affected protein features as well as splice sites, which were considered to have a disease-causing effect.

When compared the structures of WT PHEX protein with those of novel missense mutations, we found that the entire structure of C142W substitutions revealed relatively loose and several altered secondary structure sites (Supplementary Fig. S6A) (16). Moreover, the appearance of an indole ring in the carbon chain had also changed the amino acid from polar to nonpolar, resulting in changes of interactions and molecular distance with adjacent amino acid. The spatial structure of Q189H variant showed relatively compact and some secondary structure sites were also altered in the transmembrane region. In terms of amino acid properties, the original neutral polarity was changed to a positively charged one; ie, the C-H bond had transformed into imidazole ring (Supplementary Fig. S6B) (16), which eventually changed the charge distribution and protein stability. As for L450P variant, the secondary structure of PHEX was also altered significantly. Not only were some α-helices substituted by β-sheets, the appearance of nitrogen heterocycle also changed molecular interactions and protein hydrophobicity (Supplementary Fig. S6C) (16).

### Sex Differences and Genotype-phenotype Correlation in XLHR Patients

No sex differences for clinical phenotype were observed in our XLHR patients (Table 2). Next, we subdivided patients into 2 groups according to different criteria: truncated/nontruncated variants and N-terminal/C-terminal variants (9), respectively. However, age of disease onset, height SDS, serum Pi, ALP, PTH, 25-(OH)D, FGF23, and αKlotho levels at diagnosis showed no significant phenotypic differences between these 2 groups in our cohort (Table 3).

**Table 2.** Sex difference of clinical phenotypes in our XLHR cohort

	Male	Female	*P-*value
Age of disease onset (months)	15 (12, 24) n = 22	15 (12, 21) n= 21	.54
Height SDS	−2.22 ± 1.35 n = 20	−2.47 (−3.11, −1.49) n = 20	.95
Serum phosphate (mmol/L)	.83 ± 0.19 n = 22	0.79 ± 0.11 n = 21	.34
Serum ALP (U/L)	716.71 ± 274.06 n = 22	709.62 ± 241.64 n = 21	.82
Serum PTH (pg/mL)	84.61 ± 32.61 n = 18	64.24 (51.73, 121.63) n = 18	.64
Serum FGF23 (ng/L)	1180.34 (819.60, 1324.66) n = 14	1063.64 (900.60, 1184.87) n = 14	.46
Serum αKlotho (ng/L)	15.64 (13.21, 16.92) n = 14	16.55 ± 5.37 n = 14	.71

Abbreviations: FGF23, fibroblast growth factor 23; SDS, SD score; XLHR, X-linked hypophosphatemia.

**Table 3.** Genotype–phenotype correlation in our XLHR patients

	Truncated mutations	Nontruncated mutations	*P*-value	N-terminal mutations (from 5′ end to 649AA)	C-terminal mutations (from 650AA to 3′ end)	*P*-value
Age of disease onset (months)	17.64 ± 8.59 n = 25	15 (12, 32) n = 15	.90	16 (12, 24) n = 32	12 (12, 18) n = 10	.16
Height SDS	−2.07 (−3.06, −0.68) n = 23	−2.38 ± 1.53 n = 14	.77	−2.42 ± 1.25 n = 32	−2.52 ± 3.62 n = 8	.94
Serum phosphate (mmol/L)	0.81 ± 0.17 n = 25	0.73 ± 0.23 n = 16	.20	0.81 ± 0.16 n = 32	0.77 ± 0.14 n = 10	.41
Serum ALP (U/L)	707.96 ± 236.39 n= 25	741.67 ± 230.23 n = 15	.66	742.97 ± 266.38 n = 32	640.9 ± 224.07 n = 10	.28
Serum PTH (pg/mL)	83.8 ± 41.30 n = 22	95.68 ± 52.02 n = 13	.46	70.68 (54.94, 108.65) n = 25	74.84 ± 27.17 n = 10	.56
Serum FGF23 (ng/L)	1090.95 (986.43, 1223.07) n = 19	1061.86 ± 291.83 n = 9	.56	1109.47 ± 286.57 n = 21	1943.24 ± 2312.97 n = 7	.53
Serum αKlotho (ng/L)	17.15 ± 3.93 n = 19	6.09 ± 3.93 n = 9	.41	16.07 ± 4.25 n = 21	17.97 ± 8.5 n = 7	.88

Abbreviations: ALP, serum alkaline phosphatase; FGF23, fibroblast growth factor 23; SDS, SD score; XLHR, X-linked hypophosphatemia.

### Functional Analysis

#### Expression of WT and mutant PHEX proteins in vivo and in vitro

**Figure 2.**
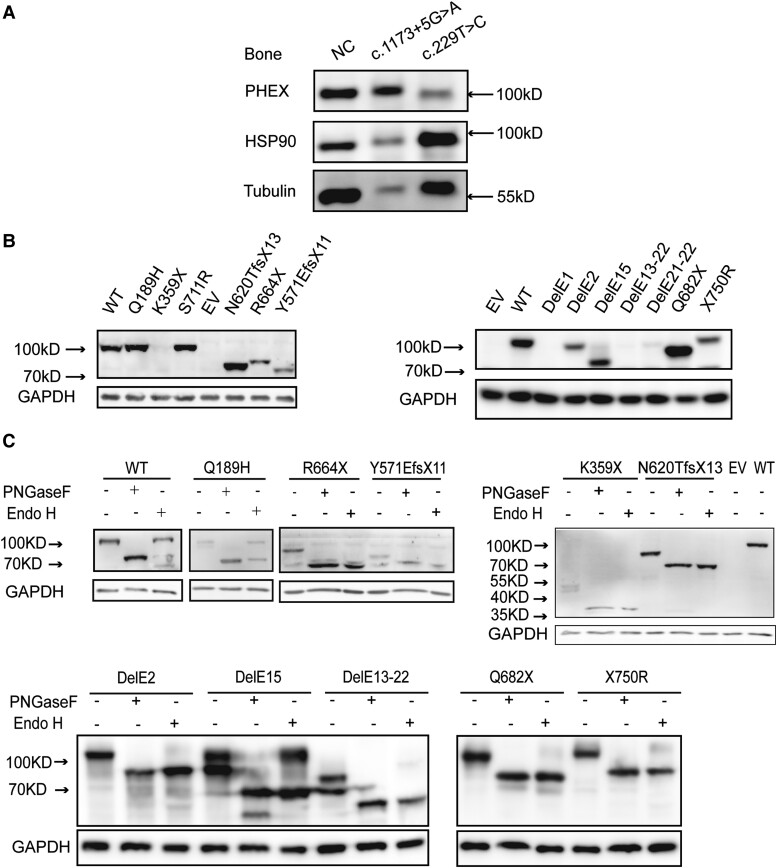
Expression and glycosylation patterns of WT and mutant PHEX proteins in vivo and in vitro. (A) The lysates of bone tissues from normal control and 2 XLHR patients were fractioned on 10% SDS-PAGE and analyzed by immunoblotting with an anti-PHEX antibody. Patient 1 carried the c.1173 + 5G > A variant and patient 2 carried the c.229T > C variant. (B) Whole cell lysates from HEK293 cells transiently transfected with WT or mutant PHEX plasmids were fractioned on 10% SDS-PAGE and analyzed by immunoblotting with anti-PHEX antibody. (C) Whole cell lysates from HEK293 cells transiently transfected with WT or mutant PHEX plasmids were incubated either with buffer, PNGase F, or endo H. The cell lysates were then fractioned on 10% SDS-PAGE and analyzed by immunoblotting with an anti-PHEX antibody. The upper band represents the fully glycosylated mature form whereas the lower bands represent a core glycosylated or deglycosylated form. Abbreviations: PHEX, phosphate regulating endopeptidase homolog X-linked; WT, wild-type; XLHR, X-linked hypophosphatemia.

To further explore the pathogenic role of novel *PHEX* variants, we performed a series of experiments. First, we evaluated the protein expression pattern of PHEX in bone tissues from 2 XLHR patients who had undergone surgeries, 1 carrying a splicing variant (c.1173 + 5G > A) and the other carrying a missense variant (c.229T > C, p.C77R). We found that the expression of C77R mutant was significantly decreased compared with normal control whereas PHEX expression of another XLHR patient was increased, indicating that *PHEX* variants could contribute to disease progression by influencing the expression of PHEX (Fig. 2A). In addition, the expression of heat shock protein 90, which was associated with conformational maturation of most client proteins and cellular homeostasis (11), was obviously increased in the patient carrying the c.229T > C variant, supporting the fact that pathogenic *PHEX* variants could abort the folding process of PHEX.

Next, we tested the overall expression levels of WT and mutant PHEX in HEK293 cells transfected with vectors expressing Flag-WT-PHEX or Flag-mutant-PHEX. Immunoblot analysis showed that WT PHEX was presented as a single band with an approximate molecular weight of 100 kDa and Q189H together with S711R mutations situated at the same position with WT (Fig. 2B). The X750R mutant protein showed a slightly lengthened protein above 100 kDa. In contrast, 8 mutants including N620TfsX13, R664X, Y571EfsX11 (Fig. 2B, left) and EX2del, EX15del, EX21-22del, Q682X (Fig. 2B, right) were shown as truncated proteins with smaller protein sizes, indicating them as premature termination of frameshift variants. In addition, most of the new mutants showed a remarkable decrease in the levels of protein expression, whereas mutations (N620TfsX13, Q682X) resulted in a significant increase in protein expression as compared to the WT protein. Notably, we did not observe proteins generated from the K359X, EX1del, EX13-22del variant below 70 kD. The proteins could either be too weak to be detected or smaller than 70 kD.

#### Glycosylation pattern of WT and mutant PHEX proteins

To determine the molecular basis accounting for the differential PHEX expression and to determine whether the lower molecular weight band represented immature PHEX, a deglycosylation experiment was performed for protein maturation analysis with 2 kinds of glycosylases, PNGase F and Endo H. This treatment could distinguish between core-glycosylated proteins present in the endoplasmic reticulum and fully glycosylated proteins with completely processed form through the Golgi apparatus. Both WT and new mutants were completely sensitive to the digestion with PNGase F, and a single protein band of 85 kDa was observed after removing N-linked oligosaccharide side chains (Fig. 2C). With regard to the Endo H-treated group, most of the WT protein remained unaltered and mutants (Q189H and EX15del) were partially resistant to Endo H digestion (approximately 50%). In contrast, R664X, Y571EfsX11, K359X, N620TfsX13, EX2del, EX13-22del, Q682X, and X750R mutants were completely susceptible to Endo H digestion.

#### Cellular localization of WT and mutant PHEX proteins

**Figure 3.**
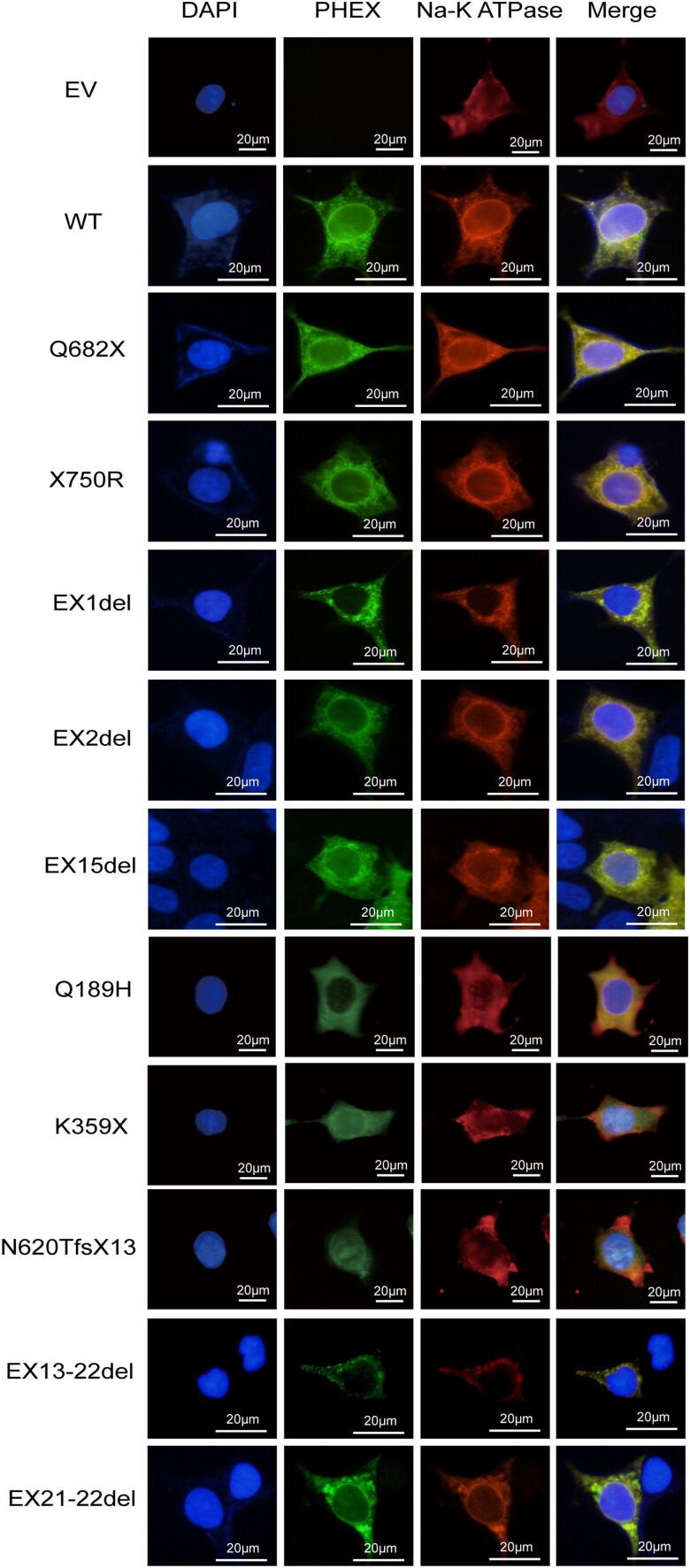
Subcellular localization analysis of WT and PHEX mutants in HEK293 cells. HEK293 cells were transfected with various PHEX plasmids carrying WT-PHEX or mutated PHEX. Forty-eight hours later, cells were fixed, permeabilized, and immunostained with anti-PHEX antibody and anti-Na^+^/K^+^ ATPase (cell membrane marker) antibody. Nuclei were visualized by 4′6-diamidino-2-phenylindole. The slides were visualized on a fluorescence microscopy (Carl Zeiss, Germany). Scale bars represent 20 μm. Abbreviations: PHEX, phosphate regulating endopeptidase homolog X-linked; WT, wild-type.

To determine whether novel mutations altered PHEX subcellular localization, an immunofluorescence assay was performed. The results showed that both WT and the mutants were detected in permeabilized HEK293 cells, after co-immunostaining with Na-K-ATPase, a cell membrane marker, WT PHEX together with variants (Q189H, N620TfsX13, Q682X, X750R, EX2del, EX15del) were colocalized on the cell membrane, whereas K359X, EX1del, EX13-22del, and EX21-22del mutants were partially colocalized with Na-K-ATPase, resulting in intracellular retention of the protein (Fig. 3).

#### **Endopeptidase activity of secPHEX proteins and PHEX enzymatic activity**–**phenotype correlation analysis in XLHR patients**

**Figure 4.**
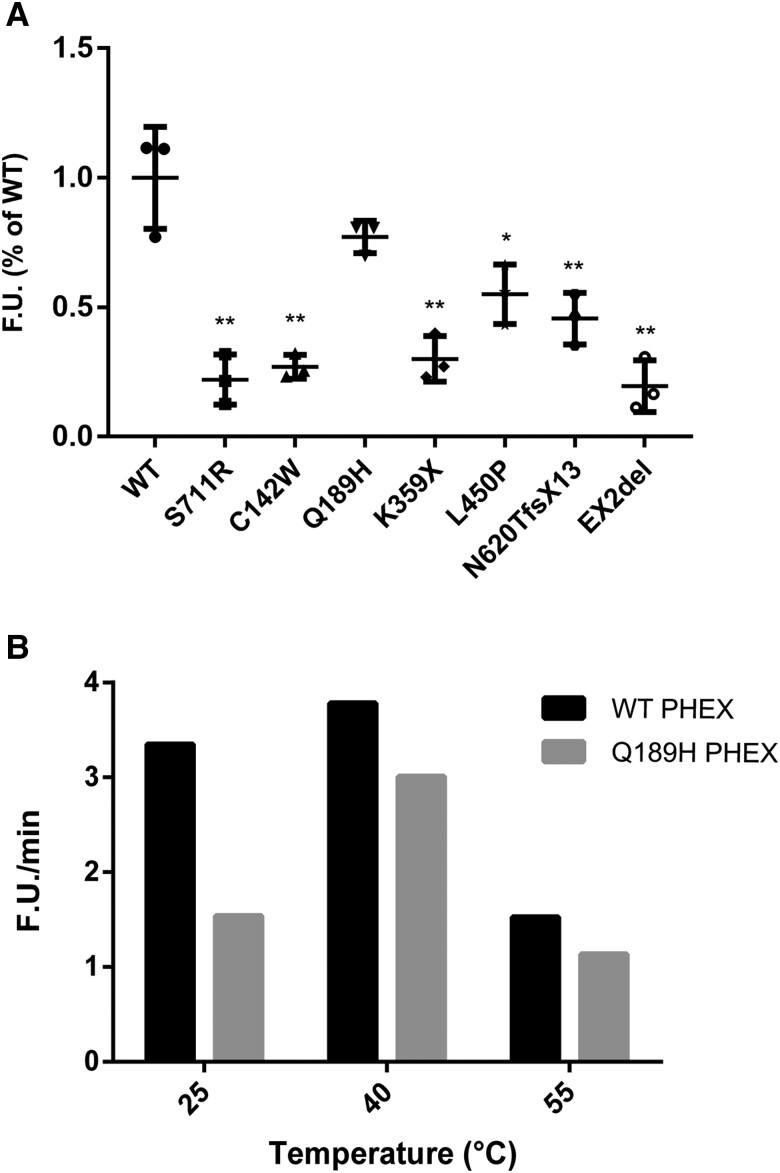
Endopeptidase activity of WT and mutant secPHEX proteins. (A) secPHEX proteins (200 ng) were incubated with fluorogenic peptide substrate Abz-GFSDYK(Dnp)-OH (30 μM) for 30 minutes and fluorescence was recorded. The amount of WT and mutant secPHEX proteins was determined by densitometry of immunoblotting. The F.U. for WT secPHEX was considered as 100%. (B) Heat stability of WT and mutant secPHEX proteins. WT and mutant secPHEX proteins (200 ng) were incubated at 25 °C, 40 °C, and 55 °C for 5 minutes before assessing the endopeptidase activity with the fluorogenic peptide substrate (30 μM). The velocity (F.U./min) was calculated from the linear portion of the curve. Bars depict the mean ± SD of 3 independent assays for each PHEX variant [n = 3, *P* = .584 (Q189H vs WT), P* = .003 (L450P vs WT), P**＜.001 (C142W/K359X/N620TfsX13/EX2del vs WT, Bonferroni's multiple comparisons test]. Abbreviations: F.U., fluorometric unit; PHEX, phosphate regulating endopeptidase homolog X-linked; WT, wild-type.

As a member of the M13 subfamily, PHEX resembles neutral endopeptidase when functioning in the form of enzyme; thus we determined the catalytic activity of secPHEX. Media from HEK293 cells expressing Y571EfsX11, R664X, Q682X, X750R, EX15del, EX21-22del mutants were devoid of secPHEX protein (data not shown). As shown in Fig. 4A, we found that except Q189H, other mutants (C142W, K359X, L450P, N620TfsX13, EX2del) exhibited a significantly decreased activity relative to WT secPHEX activity, which showed a reduction of ∼27%, 25%, 55%, 45%, and 20%, respectively. S711R is a known pathogenic variant, and we used it as a positive control (12). Although the Q189H variant only showed a slight decrease in its enzyme activity, it appeared unstable while testing the enzyme activity. When treated in 25 °C, the variant exhibited only 50% of WT activity (Fig. 4B).

**Figure 5.**
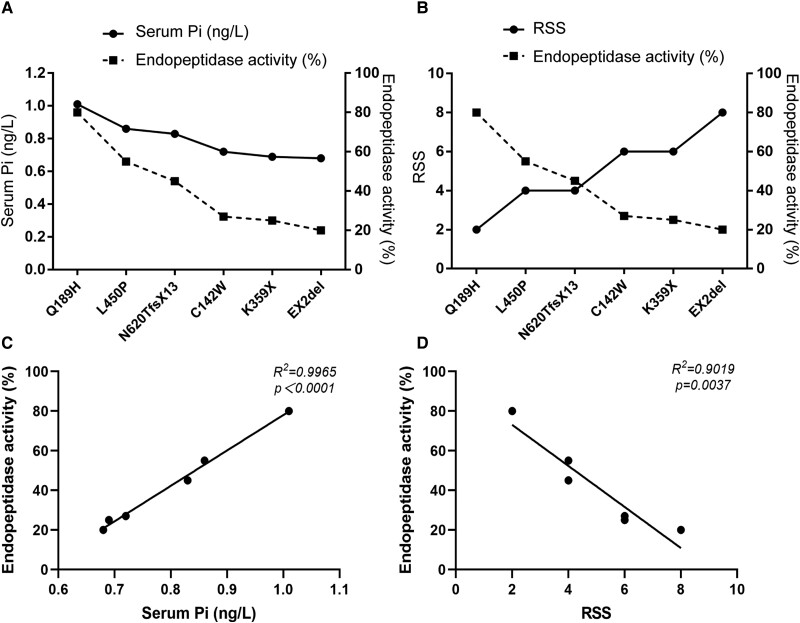
Endopeptidase activity-phenotype correlation analysis in our XLHR patients. (A) The correlation between serum phosphorus level (mmol/L) and endopeptidase activity (%) of secPHEX. (B) The correlation between RSS of XLHR patients and endopeptidase activity (%) of secPHEX. Abbreviations: PHEX, phosphate regulating endopeptidase homolog X-linked; RSS, Rickets Severity Score; XLHR, X-linked hypophosphatemia.

In addition, we explored the relationship between indicators of disease severity of XLHR patients, such as serum Pi, RSS, and enzymatic activity of PHEX. As shown in Fig. 5, serum Pi levels had a positive correlation with PHEX enzymatic activity: a low level of phosphorus accompanied by a relatively low catalytic activity of PHEX. Because RSS had a negative correlation with PHEX enzymatic activity, it implicated that patients with a lower RSS often had highly active PHEX. For example, the EX2del mutant presented the lowest catalytic activity as presented earlier, which was consistent with the lowest serum Pi level (0.68 mmol/L) and highest RSS (8 points) of the patient carrying this variant.

#### *Phex*-T1349C and *Phex*-C426G mice developed clinically relevant phenotypes of XLHR in an enzymatic activity-dependent manner

**Figure 6.**
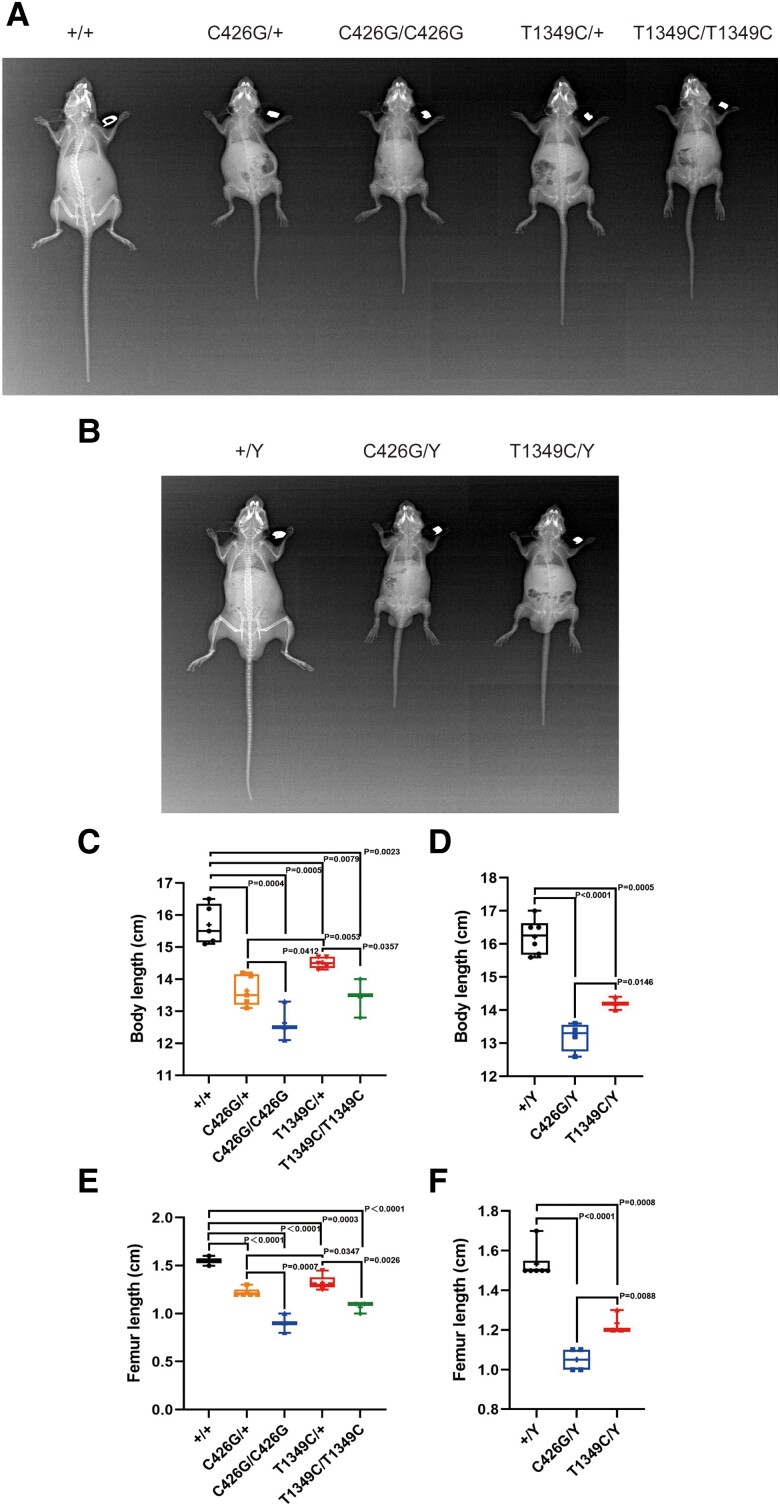
Growth characteristics of *Phex*-C426G and *Phex*-T1349C knock-in mice. (A) Faxitron X-ray images of WT and *Phex*-C426G at 12 weeks of age. (B) Faxitron X-ray images of WT and *Phex*-T1349C mice at 12 weeks of age. (C) Body length of age-matched (12-week-old), sex-matched mutant and WT mice. (D) Femoral length of age-matched (12-week-old), sex-matched mutant and WT mice. Significant differences of different genotypes are expressed with horizontal bars and corresponding *P*-values by Student's *t*-test for normally distributed continuous variables with homogeneous variance or Mann–Whitney test for nonnormally distributed continuous variables with nonhomogeneous variance. Abbreviations: WT, wild-type.

To further characterize PHEX enzymatic activity–phenotype correlation analysis, we selected 2 missense variants (c.C426G and c.T1349C) and generated 2 new knock-in XLHR mouse models using CRISPR/Cas9 technology. Sequencing of the *Phex* gene of the mutant mice confirmed both mouse models carried the corresponding mutation sites (Supplementary Fig. S8) (16). Both hemizygous males (*Phex*^C426G^/Y*, Phex*^T1349C^/Y) and heterozygous or homozygous females (*Phex*^C426G^/+, *Phex*^T1349C^/+, *Phex*^C426G^/*Phex*^C426G^, *Phex*^T1349C^/*Phex*^T1349C^) developed typical XLHR phenotypes from 3 weeks of age (data not shown), and their gross appearance included a smaller body size and shortened hind limbs and tail. In addition, X-ray analysis of both male and female mice at 12 weeks of age clearly detected skeletal aberrations consistent with rickets, including severe osteopenia of the whole skeleton, shortened long bone, abnormal curvature of pelvic and long bones, and dental dysplasia (Fig. 6A and 6B). Moreover, both variants were fully penetrant because affected males did not sire unaffected females. Furthermore, compared with age- and sex-matched *Phex*^T1349C^ mice, *Phex*^C426G^ mice showed more severe phenotypes including less weight and shorter limbs (Fig. 6C–6F).

**Figure 7.**
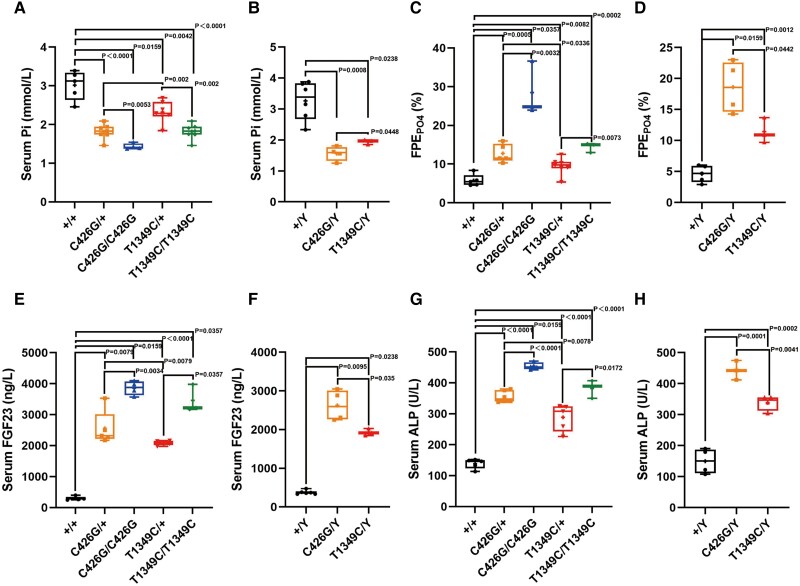
Serum biochemistry and urine phosphate excretion of *Phex*-C426G and *Phex*-T1349C mice. Serum phosphorus (A, B), urine fractional phosphate excretion (FPEPO4) (C, D), serum FGF23 (E, F), as well as alkaline phosphatase levels (G, H). Samples were collected at sacrifice from age-matched (12-week-old), sex-matched *Phex*-C426G, *Phex*-T1349C, and WT mice. Significant differences between animals of different genotypes are reported above the data expressed with horizontal bars and corresponding *P*-values by Student's *t*-test for normally distributed continuous variables with homogeneous variance or the Mann–Whitney test for nonnormally distributed continuous variables with nonhomogeneous variance. Abbreviations: WT, wild-type.

One hallmark of XLHR is hypophosphatemia; thus we next analyzed serum parameters related to phosphate metabolism. The results showed that Pi levels were significantly lower (Fig. 7A and 7B), whereas urine fractional excretion of phosphorus (Fig. 7C and 7D), serum FGF23 (Fig. 7E and 7F), and ALP (Fig. 7G and 7H) levels were significantly elevated in mutant hemizygous males and heterozygous/homozygous females vs age- and sex-matched WT mice. A clear gene–dose effect was observed. Consistently, *Phex*^C426G^ mice had lower serum Pi levels, higher fractional excretion of phosphorus, and higher ALP and FGF23 levels.

**Figure 8.**
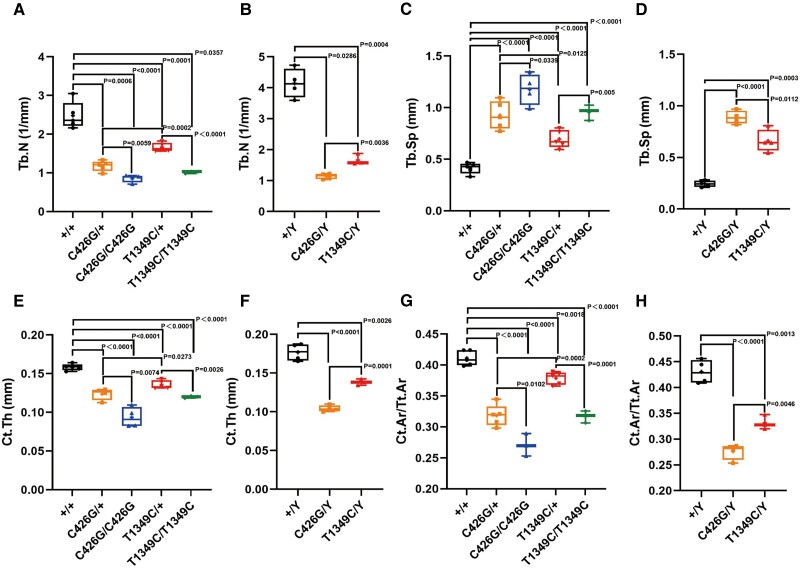
Microcomputed tomography analysis of the femurs of *Phex*-C426G, *Phex*-T1349C mice, and WT littermates. Trabecular bone parameters include Tb.N (A, B) and Tb.Sp (C, D) in female and male mice. Cortical bone parameters include Ct.Th (E, F) and Ct.Ar/Tt.Ar (G, H) in female and male mice. Significant differences between animals of different genotypes are reported above the data expressed with horizontal bars and corresponding *P*-values by Student's *t*-test for normally distributed continuous variables with homogeneous variance or the Mann–Whitney test for nonnormally distributed continuous variables with nonhomogeneous variance. Abbreviations: Ct.Ar/Tt.Ar, cortical bone area/cortical total area; Ct.Th, cortical thickness; Tb.N, trabecular number; Tb.Sp, trabecular separation; WT, wild-type.

Another hallmark of XLHR is rickets/osteomalacia, which prompted us to perform bone histomorphometric analysis. We found that at distal femoral metaphysis, both *Phex*^T1349C^ and *Phex*^C426G^ mice had significantly less trabeculae (Fig. 8A and 8B) and greater trabecular separation (Fig. 8C and 8D) in both males and females compared to WT littermates. At mid-diaphysis of the femur, *Phex*^T1349C^ and *Phex*^C426G^ mice had significantly less cortical bone area/cortical total area and cortical thickness (Fig. 8E–8H) in both males and females compared to WT littermates. As expected, *Phex*^C426G^ mice demonstrated significantly severe phenotypes compared with *Phex*^T1349C^ mice. Consistent with skeletal abnormalities of XLHR, both knock-in mice had significantly less peak load and bone stiffness compared to WT littermates while *Phex*^C426G^ mice showed increased bone fragility (Supplementary Fig. S9) (16).

## Discussion

Herein, our present study identified 36 different *PHEX* variants in 44 XLHR probands and 5 non-*PHEX* variants, of which 22 variants were novel for the first time. In silico analysis suggested a strong possibility for pathogenic significance among all the mutations. Functional studies validated that *PHEX* variants likely contributed to the pathogenesis of XLHR through impairing protein expression, protein subcellular localization, and enzyme activity. In addition, we analyzed the relationship between catalytic activity of PHEX and clinical phenotype of XLHR patients for the first time in the XLHR research field. Furthermore, we established 2 novel knock-in XLHR mouse models by introducing c.C426G and c.T1349C variants into mice, respectively, using CRISPR/Cas9 technology. Our in vivo studies using these 2 mouse models demonstrated that typical clinical manifestations of XLHR occurred in a gene–dosage and Phex activity-dependent manner, including growth retardation, skeletal dysplasia (rickets/osteomalacia), and hypophosphatemia, which was accompanied by elevated serum FGF23 and ALP levels. Taken together, our bioinformatic analysis and functional analysis provided compelling evidence for the pathogenic function of those newfound PHEX mutations.

The primary diagnosis of our patients presented a typical clinical phenotype of XLHR including short stature, lower limb deformities, and high circulation levels of FGF23 and αKlotho, consistent with previously published studies (7, 17-20). Most of the PHEX variants (25/40, 62.5%) were predicted to produce truncated PHEX proteins, including nonsense variants, frameshift variants, and large insertions/deletions. The genotype–phenotype correlation of XLHR still remains controversial. In our cohort, no significant genotype–phenotype association was observed between different mutation types and disease severity such as age of onset, height SDS, serum Pi, ALP, PTH, 25-(OH) D3, FGF23, and αKlotho level, which was in agreement with previous studies (5-9). It is possible that other unidentified factors may contribute to the severity of hypophosphatemic rickets independent of *PHEX* gene alteration.

It is generally accepted that truncated mutations tend to result in more severe dysfunction, while the missense mutants may retain partial function leading to a milder phenotype (21). In our cohort, we characterized protein expression, glycosylation, subcellular localization, and endopeptidase activity of different types of novel PHEX variants and found no significant differences between missense and nonsense variants, which was in accordance with previous studies (5, 6). Most of the novel *PHEX* variants significantly impaired the synthesis of PHEX except for 2 truncating variants N620TfsX13 and Q682X, but these 2 mutants were underglycosylated, which likely contributes to disrupted protein function. Moreover, except for 1 nontruncating variant (Q189H) and 1 truncating variant (EX15del), the majority of variants produced immature proteins that were not fully glycosylated. Although most mutants localized to the cell membrane, the cellular retention of some truncating variants (K359X, EX1del, EX13-22del, and EX21-22del) suggested that there might an impaired trafficking and endoplasmic reticulum retention consistent with previous studies (5, 22). In addition, some mutant secPHEX could not be secreted to the media, which implied that those mutations might have a strong impact on maintaining the structure of PHEX.

Because PHEX is a transmembrane endopeptidase belonging to the type II integral membrane zinc-dependent endopeptidase family, we speculated that PHEX might regulate phosphorus metabolism through catalyzing its substrates. To validate this hypothesis, we performed an enzyme activity-phenotype analysis that has never been done before and was the major innovative point of our study. As expected, the levels of serum Pi of XLHR patients were positively correlated with PHEX activity. Similar results were observed in the 2 novel knock-in XLHR mouse models we generated in this study. These findings strongly suggested that PHEX acted as a peptidase to regulate phosphorus metabolism via activating or inactivating biologically active peptides to induce downstream pathways. The specific molecular mechanism of PHEX in the regulation of phosphorus metabolism and bone mineralization remains unclear (23, 24). Little is known about how loss of PHEX enzymatic activity affects the proteolytic process of bone matrix proteins known to regulate mineralization. Therefore, further mechanistic studies are required to identify specific components involved in PHEX-mediated proteolysis of bone matrix proteins in the future.

At present, the first-line treatment for XLHR is phosphate salts and calcitriol (25). However, their curative effects are very limited because they cannot reverse PHEX dysfunction (26, 27). The new treatment Crysvita (burosumab), a monoclonal antibody of human FGF23, has been approved in the United States and Europe, which demonstrated effectiveness for XLHR patients (28-31). Nonetheless, the clinical outcome of burosumab in Chinese XLHR patients remains unknown. In addition, long-term clinical data is lacking with regard to the incidence of hyperparathyroidism, renal function, or the growth and development of children in patients treated with burosumab. Therefore, clinical studies of burosumab in Chinese XLHR patients are urgently needed in order to develop effective therapeutic approaches for the treatment of XLHR patients in China and other Asian countries. Also, we anticipated that our XLHR cohort would provide important information about the clinical outcomes of burosumab in China. Our future goals are to continue to collect samples in patients with XLHR for a more robust genotype–phenotype analysis and to further characterize how some of the mutants identified in our study affect other signaling pathways using our new knock-in mouse models.

In summary, our study represents one of the most comprehensive functional characterization of *PHEX* gene variants identified in a new cohort of XLHR patients. Our group was also the first to establish peptidase activity–phenotype correlation. Our data expand the mutation spectrum of *PHEX* related to XLHR and facilitates the understanding of XLHR pathogenesis. Our clinical cohort also provides valuable resources to explore new therapeutic strategies in future studies.

## Data Availability

Original data generated and analyzed during this study are included in this published article or in the data repositories listed in References.
